# The Terminations of the Olfactory Nerve

**Published:** 1850-12

**Authors:** 


*The Terminations of the Olfactory Nerve.*—Dr. Hom describes the olfactory nerves as being easily seen by taking a piece of a frog's nasal mucous membrane, placing it between glass, and examining it with a power of from +216 to +300. The extremities of the nerve-fibres, sometimes dilated and forming club-shaped processes, can be seen at their extremities, winding round and sometimes returning to the parent branch. Besides these, other nerves, with broad diameters, are seen passing in various directions, and present the characters of fine cerebral fibres.—*Ibid, from Mulier's Archiv.*

